# Impact of *Helicobacter pylori* infection on iron deficiency anemia in children: a systematic review and meta-analysis with early intervention implications

**DOI:** 10.3389/fmicb.2025.1541011

**Published:** 2025-06-19

**Authors:** Ziteng Wang, Wentao Tan, Huanhuan Xiong, Jiali Huang, Herui Wei, Mengqi Li, Jing Luo, Wen An, Lingling He, Jiali Ma, Fan Xiao, Hongshan Wei

**Affiliations:** ^1^Department of Gastroenterology, Beijing Ditan Hospital, Capital Medical University, Beijing, China; ^2^Department of Gastroenterology, Beijing Shijitan Hospital, Capital Medical University, Beijing, China; ^3^Department of Rheumatology and Immunology, Zhumadian Central Hospital, Zhumadian, Henan, China; ^4^Department of Gastroenterology, Peking University Ditan Teaching Hospital, Beijing, China; ^5^National Key Laboratory of Intelligent Tracking and Forecasting for Infectious Diseases, Beijing Ditan Hospital, Capital Medical University, Beijing, China; ^6^Beijing Key Laboratory of Emerging Infectious Diseases, Institute of Infectious Diseases, Beijing Ditan Hospital, Capital Medical University, Beijing, China; ^7^Beijing Institute of Infectious Diseases, Beijing, China; ^8^National Center for Infectious Diseases, Beijing Ditan Hospital, Capital Medical University, Beijing, China

**Keywords:** *Helicobacter pylori*, iron-deficiency anemia, children, iron deficiency, iron store

## Abstract

**Background:**

This study investigates the association between *Helicobacter pylori* (*H. pylori*) infection and iron deficiency (ID) as well as its potential link to iron deficiency anemia (IDA) in children.

**Methods:**

As of August 2024, we conducted a comprehensive literature review using the Embase, PubMed, Web of Science, Ovid Medline, and Cochrane databases to compare the risk of IDA in patients with and without *H. pylori* infection. The Newcastle-Ottawa Quality Assessment Scale was used to assess the methodological quality of the included studies. Odds ratios (ORs) and 95% confidence intervals (CIs) were extracted from the studies, and data were transformed for meta-analysis. Heterogeneity was evaluated using the I^2^statistic, and homogeneity was tested with the chi-square test. If the I^2^ value was 50% or greater, a random-effects model was applied; if the I^2^ value was below 50%, a fixed-effects model was used. The meta-regression was performed to explore the potential sources of heterogeneity. Additionally, a meta-analysis was conducted to assess the changes in hemoglobin and ferritin concentrations before and after *H. pylori* eradication.

**Results:**

We analyzed data from patients across 13 countries. Our findings reveal that individuals infected with *H. pylori* have a higher likelihood of developing ID compared to those uninfected, with an OR = 1.52 (*p* < 0.00001, 95% CI 1.32–1.74, I^2^ = 44%). The risk of IDA is also higher in patients with *H. pylori*, with an OR = 1.83 (*p* = 0.05, 95% CI 1.01–3.33, I^2^ = 67%). Conversely, the overall effect of *H. pylori* infection on anemia is minimal, with an OR = 0.94 (*p* = 0.64, 95% CI 0.73–1.21, I^2^ = 96%). Meta-regression results suggest that age is the main source of heterogeneity. A meta-analysis of treatment-related randomized controlled trials revealed that combining iron supplementation with *H. pylori* eradication therapy significantly raised ferritin levels, with an SMD (Standardized Mean Difference) = 0.86 (*p* = 0.02, 95% CI 0.14–1.57, I^2^ = 89%). Hemoglobin levels also showed an increase, with an SMD = 0.47 (*p* = 0.04, 95% CI 0.01–0.93, I^2^ = 75%).

**Conclusion:**

In children, there is a significant association between *H. pylori* infection and both ID and IDA. Additionally, the eradication of *H. pylori* has been shown to lead to an improvement in iron stores.

**Systematic review registration:**

https://www.crd.york.ac.uk/PROSPERO/display_record.php?RecordID=426395. PROSPERO ID: CRD42023426395.

## 1 Introduction

### 1.1 *Helicobacter pylori* overview

The gastrointestinal tract is home to millions of microorganisms that collectively form the intestinal microbiota, interacting with the host to maintain homeostasis. Among these microorganisms, *Helicobacter pylori* (*H. pylori*) is one of the most prevalent and widely studied bacteria. The World Health Organization (WHO), in 1994, classified *H. pylori* as a class I carcinogen due to its strong association with gastric cancer (Blanchard and Czinn, 2017). *H. pylori* is a microaerophilic, gram-negative bacterium that colonizes the gastric mucosa in a spiral configuration. Humans are the exclusive host and reservoir for this bacterium, and its prevalence is particularly high in socioeconomically disadvantaged regions, with infection rates exceeding 80% in some areas (Coelho et al., 2018). *H. pylori* disrupts the gastric mucosal barrier and produces vacuolating cytotoxin, which contributes to the pathogenesis of various gastrointestinal diseases, including peptic ulcer disease and gastric cancer (Malfertheiner et al., 2012). In recent years, studies have also identified extra-gastrointestinal manifestations of *H. pylori* infection, such as iron deficiency anemia (IDA) (Kato et al., 2022), vitamin B12 deficiency, and idiopathic thrombocytopenic purpura (Robinson and Atherton, 2021).

### 1.2 Anemia as a global health concern

Anemia is also recognized by the WHO (Zinebi et al., 2017) as one of the top ten global health issues, affecting approximately one-third of the world’s population, with more than half of these cases attributed to IDA (Lopez et al., 2016). In 2016, anemia affected 41.7% of children under 5 years old, 40.1% of pregnant women, and 32.5% of non-pregnant women globally (WHO, 2022a,b). The WHO has prioritized anemia as a global health issue, aiming to reduce its prevalence among women by 50% by 2025. The clinical manifestations of anemia are primarily due to the reduced capacity of blood to carry oxygen. This reduction is influenced by changes in total blood volume and the respiratory system’s compensatory mechanisms. The most common systemic symptom of anemia is fatigue. Typical signs include pallor of the skin and mucous membranes, dyspnea, loss of appetite, and changes in urine output. Additional manifestations can include pica, alopecia, nail pitting, and atrophic glossitis (Ganz, 2019; Di Angelantonio et al., 2017). Iron deficiency (ID), characterized by reduced systemic iron levels, is the most common micronutrient deficiency worldwide. It is also the primary cause of IDA. Populations at higher risk for ID include children, premenopausal women, and individuals in low-and middle-income countries (GBD 2016 Disease and Injury Incidence and Prevalence Collaborators, 2017; Santos et al., 2020). ID disrupts oxygen transport and impairs enzymatic reactions in various metabolic pathways, leading to decreased hemoglobin levels and, ultimately, anemia.

### 1.3 Link between *Helicobacter pylori* and IDA

*H. pylori* infection and its relationship with IDA involve complex and multifactorial mechanisms (Marignani et al., 1997; Barabino et al., 1999). Firstly, *H. pylori* infection induces gastric mucosal inflammation and disrupts gastric acid secretion (Sato et al., 2015; Annibale et al., 2020; Kishikawa et al., 2020). Gastric acid is essential for the dissolution and absorption of non-heme iron (Flores et al., 2017; Flores et al., 2015), and *H. pylori* infection often leads to hypochlorhydria or reduced gastric acid secretion, hindering the conversion of dietary iron into its absorbable form (Capurso et al., 2001; Annibale et al., 2003). In addition to impairing iron absorption, *H. pylori* itself competes with the host for iron, which is crucial for its growth and survival. In a low-acid environment, *H. pylori* utilizes specialized iron uptake systems to extract iron from the host (Yokota et al., 2013; Lee et al., 2009). This competition depletes the host’s iron reserves and prompts the host to increase the synthesis of hepcidin, further suppressing iron absorption and transport (Mendoza et al., 2019). However, *H. pylori* has evolved mechanisms to evade immune surveillance, enabling it to continuously extract iron, further reducing iron bioavailability and eventually leading to the onset of IDA.

Previous studies (Muhsen and Cohen, 2008; Qu et al., 2010; Hudak et al., 2017) have largely focused on the general population and have not conducted in-depth research specifically on the pediatric population. The benefits of *H. pylori* eradication in children remain controversial. It is well known that active eradication therapy is typically not recommended for children infected with *H. pylori*. This therapeutic strategy is based on the premise that the natural course of *H. pylori* infection in children is relatively slow, and most children are able to clear the infection on their own as they grow older. However, this approach may result in some untreated children carrying *H. pylori* into adulthood, potentially increasing their risk of developing extragastric diseases, such as IDA. Currently, in children, there is a lack of comprehensive and systematic reviews or meta-analyses exploring the relationship between *H. pylori* infection and IDA. To fill this gap, we conducted a meta-analysis aimed at evaluating the association between *H. pylori* infection and IDA, exploring potential links, and elucidating the clinical value of early eradication of *H. pylori*. Therefore, whether eradicating *H. pylori* can bring benefits to the pediatric population becomes the central focus of our research. Our study emphasizes the widespread health impacts of *H. pylori* infection, particularly in children, where early intervention and eradication strategies are crucial. Through effective eradication measures, the incidence of systemic diseases, including IDA, may be prevented or significantly reduced, ultimately improving the overall health of children.

## 2 Materials and methods

Our research encompassed cross-sectional studies, cohort studies, case–control studies, as well as intervention studies and clinical trials. We conducted a meta-analysis in accordance with the PRISMA guidelines for systematic reviews and meta-analysis. Since no original clinical raw data were used in this meta-analysis, ethical approval was not required. Our research protocol (PROSPERO ID: CRD42023426395) is registered with the International Prospective Register of Systematic Reviews.

### 2.1 Search strategy and inclusion criteria

As of August 9, 2024, a comprehensive search was conducted by four independent authors (WZT, TWT, XHH, and HJL) to identify relevant English-language publications. The search covered the following databases: OVID Medline, Cochrane Library, PubMed, EMBASE, and Web of Science. The focus of the search was on studies reporting the prevalence of *H. pylori*, IDA, ID and anemia in children and adolescents aged 0 to 20 years. The search was performed using the following keywords: “iron deficiency,” “anemia,” “iron deficiency anemia,” “hemoglobin,” “ferritin,” “iron reserves,” “*Helicobacter pylori*,” “*H. pylori*” and “campylobacter pylori,” “children,” and “child.” Examples of the search strategies are provided in Appendix 1–5.

Studies were excluded based on the following criteria: (1) duplicate reports, case reports or series, letters to the editor, comments, or author responses; (2) studies that did not meet the inclusion criteria described below.

To ensure methodological rigor and minimize bias, studies were included if they met the following criteria: (1) data collection based on local hospital surveys; (2) inclusion of general population samples, rather than specific volunteer groups; (3) use of clear, validated blood tests to diagnose ID, IDA and anemia; and (4) confirmation of *H. pylori* infection through validated diagnostic methods such as carbon-13 breath tests, *H. pylori* stool tests, or gastric mucosal biopsy specimens.

We excluded factors that could potentially influence the accuracy of *H. pylori* testing, such as the certain use of proton pump inhibitors (PPIs), opioids, dietary modifications, physical activity, irritable bowel syndrome, gastrointestinal surgery, intestinal motility disorders, diverticulosis, systemic sclerosis, and hypothyroidism. No geographical restrictions were applied to the inclusion criteria.

### 2.2 Study selection process

As the first step, the database search was filtered to identify publications whose titles were relevant to the study hypothesis. The selected publications were then screened based on their abstracts, followed by a full-text review to assess their eligibility for inclusion. Any discrepancies or disagreements regarding eligibility were resolved through discussion and consensus among the three independent reviewers.

### 2.3 Data extraction

We developed Tables 1, 2 to systematically summarize the data from each study, including the study title, author names, publication date, participant demographics, study location, *H. pylori* infection testing methods, iron status markers, and the classifications for ID, IDA, and anemia. Additionally, we recorded the results and any adjustments made for confounding factors. For each study, we extracted the number of individuals infected with *H. pylori*, as well as the number of cases of IDA, ID and anemia. For studies reporting relative risk (RR) or odds ratio (ORs), along with their 95% confidence intervals (CIs) or *p*-values, we used these data directly from the original articles. When these statistics were not provided, we applied appropriate statistical methods to calculate them. In randomized controlled trials (RCTs), we extracted data on the number of participants in both the intervention and control groups, and recorded the mean and standard deviation (SD) of ferritin and hemoglobin levels at both baseline and follow-up.

**Table 1:** Studies on the association between *H. pylori* and iron stores.

Study	Location	Study design	Participants	*H. pylori* detection	Outcomes	Adjustment for confounders	NOS score
Haghi-Ashtiani et al. (2008)	Iran	Case–control	111 boys and 98 girls with a median age of 7.1 years.	Rapid urease tests, Gastric biopsy	IDA: serum ferritin level determined by ELISA was <12 mg/L and the hemoglobin level was less than adjusted values for age and sex. Anemia: severe:hemoglobin level <7 mg/dL; mild:hemoglobin level >7 mg/dL.	Age, sex	6
Mendoza et al. (2019)	Mexico	Cross-sectional study	350 schoolchildren age from 6 to14 years old	Rapid urease examination, gastric biopsy	Children less than 12 years: Iron deficiency: ferritin <15 μg/L; Anemia: Hb < 11.5 g/dL children aged 12 years or older: Hb < 12.0 g/dL	Age, sex, weight, body mass index	6
Zhang et al. (2022)	China	Cross-sectional study	902 Children aged 4–16 years	Positive endoscopic pathological staining, rapid urease test,^13^C-urea breath test	ID: SF < 12 μg/L for children younger than 5 years old, SF < 15 μg/L for older than 5 years old. IDA: hemoglobin <110 g/L for children younger than 5 years old, hemoglobin <115 g/L for 5–11 years old, and hemoglobin <120 g/L for 12–16 years old.	Gender, age, education level of the mother, place of residence, economic income level	7
Baggett et al. (2006)	Alaska	Randomized controlled trial	688 children 7-to 11-year-old children predominantly Alaska Native villages	^13^C-urea breath test (UBT)	ID: serum ferritin concentration <10 g/L IDA: ferritin level <10 g/L and a hemoglobin concentration <115 g/L	Age, gender, village of residence, number of household members, number of household members who were younger than 5 years, recent antibiotic use, and household water source.	7
Bazmamoun et al. (2014)	Iran	Case control study	200 children aged 2 to 16 years old, undergoing endoscopy from March 2012 to March 2013 at Besat Hospital of Hamedan	Gastric biopsy specimens	IDA: serum Ferritin less than 12 ng/mL and Hb below normal range (for the age and gender)	Body mass index	7
Baysoy et al. (2004)	Turkey	Randomized controlled trial	52 patients were eligible for the study (29 female and 23 male; 8.9 ± 4.7 years) recurrent abdominal pain, refractory IDA, and other gastrointestinal complaints	Rapid urease test, biopsy	IDA: ferritin level was lower than 12 g/L, serum iron level was lower than 50 g/dL, and the hemoglobin level was more than 2SD below age-and gender-adjusted normal values.	3-day vitamin C and iron consumption, serum gastrin levels, and gastric juice ascor-bic acid levels	8
Al-Sinani et al. (2014)	Oman	Double-blind randomized trial	143 children who underwent esophageo-gastro-duodenoscopy (EGD) during 3 years period (January 2010–January 2013) at pediatric gastroenterology unit, Department of Child Health, Sultan Qaboos University Hospital (SQUH)	Gastric biopsies	Anemia: hemoglobin level below the normal range for age of included children	Age, gender, indication for EGD	7
Muhsen et al. (2009)	Israeli Arab	Cross-sectional study	509 Israeli Arab children aged 1 to 19 years	Serum *H. pylori*–specific IgG antibodies	ID: ferritin level lower than12 mg/L	Sex, Socioeconomic index	6
DiGirolamo et al. (2007)	USA	Randomized controlled trial	86 children aged 12–71 months living in a small coastal village in southwest Alaska.	Serologic IgG, UBT, *H. pylori* stool antigen enzyme immunoassay	Anemia: hemoglobin (Hgb) concentration less than 11.0 g/dL for children less than 2 years of age, less than 11.1 g/dL for children aged 2 years to less than 5 years, and less than 11.5 g/dL for children aged 5 years.	Age and gender	6
Christofides et al. (2005)	Canada	Cross-sectional survey	115 children from one Inuit and two Cree First Nations communities participated.	*H. pylori* IgG antibodies	Anemia: Hb < 110 g/L. ID: Soluble transferrin receptor (sTfR)>8.5 mg/L.	Dietary data, age, sex	6
Pacey et al. (2011)	Canada	Cross-sectional study	Inuit (*n* = 388) aged 3–5 years randomly recruited from communities	ELISA of anti *H. pylori* IgG antibodies	Anemia (3–4 years: Hb<110 g/L; 5 years: Hb<115 g/L). ID (ferritin<12 mg/L)	Demographic information, medical history, anthropometrics,	8
Mehata et al. (2021)	Nepal	Cross-sectional survey	Under-five children (*n* = 1709), adolescents (*n* = 1865) aged 10–19 years and married non-pregnant women aged data from the Nepal National Micronutrient Status Survey 2016 (NNMSS-2016).	*H. pylori* antigen in stool samples, serum *H. pylori* specific antibody (IgG)	ID: Serum ferritin <12 mg/dL	Age	6
Abdelaziz et al. (2021)	Egypt	Cross-sectional survey	1,200 children during the academic year 2017 to 2018 in primary schools in Sharqia governorate, Egypt	*H. pylori* antigen in stool	Anemic: Hb level<11.5 g/dL	Sex, age	8
Lupu et al. (2022)	Romania	Cross-sectional survey	A group of 1757 patients aged from 6 to 12 years from 1 April 2013–31 March 2016 mainly hospitalized in the Gastroenterology Pediatric Clinic	*H. pylori* antigen in stools, Biopsies	Anemic: Hb level<11.5 g/dL	None	6
Zamani et al. (2011)	Tehran	Cross-sectional survey	1,665 primary school children (43% boys, 57% girls) with mean age 9.2 ± 1.5 years were enrolled in the study 6–12 year old healthy primary school children in Tehran during the academic year 2005–2006	ELISA of anti *H. pylori* IgG	ID: Serum ferritin <12 mg/dL	None	7
Seo et al. (2002)	Korea	Cross-sectional study	753 schoolchildren (419 boys and 334 girls, age range: 6–12 years) in an elementary school located in Seoul	*H. pylori* IgG antibody concentrations were determined using an ELISA commercial kit	ID: a serum ferritin level <15 ng/mL	Age, sex	6
Taye et al. (2015)	Ethiopia	Cohort study	In 2011/12, 856 children (85.1% of the 1,006 original singletons in a population-based birth cohort) were followed up at age six and half.	SD Bioline *H. pylori* stool antigen test (Standard Diagnostics, Inc)	Anemia was defined according to WHO hemoglobin cutoffs: < 11.5 g/dL for children 5–11 years	Place of residence, ethnicity, religion, maternal education, source of water, crowdedness, sanitary conditions, history of vaccination and history of vitamin A supplementation	7
Liu et al. (2024)	China	Clinical comparative study	126 children who were first diagnosed as *H. pylori* infection in Baoding Children’s Hospital and 200 children without *H. pylori* infection	Stool *H. pylori* antigen test and/or ^13^C-urea breath test	Routine blood test, serum ferritin (SF), serum iron (SI) and total iron binding capacity (TIBC)	None	7

**Table 2:** Clinical trials assessing the impact of anti-*H. pylori* therapy on hemoglobin and iron biomarkers.

Study	Country	Participants with the following conditions	Age	Design	*H. pylori*	Intervention	Control	Outcomes
Choe et al. (1999)	South Korea	IDA & *H. pylori* infection	10–17	RCT	Rapid urease test and histology	*H. pylori* treatment and oral iron therapy; Placebo iron and eradication therapy;	Iron therapy and placebo	HB, ferritin, TIBC
Xia et al. (2012)	China	IDA & *H. pylori* infection	12–18	RCT	Serum *H. pylori* IgG antibodies and stool antigen EIA	12-week course of oral DTA–Na–Fe and a 2-week course of colloidal bismuth subcitrate, amoxicillin and etronidazole	12-week course of oral EDTA–Na–Fe alone	Hb, serum, transferrin receptor, ferritin
Cardenas et al. (2011)	USA	*H. pylori* infection	3–10	RCT	Urine based rapid test and ^13^C urea breath test (UBT)	*H. pylori* eradication and iron; *H. pylori* eradication and placebo.	Placebo and iron; Placebo only.	Serum ferritin, transferrin saturation, hemoglobin.
Fagan et al. (2009)	Alaska	*H. pylori* infection & ID	7–11	RCT	^13^C-labeled urea breath test	Iron sulfate (6 week course) plus *H. pylori* treatment	Iron sulfate alone (6 week course)	HB, ferritin
Duque et al. (2010)	Mexico	*H. pylori* & ID or anemia	6–13	RCT	A ^13^C urea breath test	*H. pylori* eradication and ferrous sulfate plus folic acid	*H. pylori* eradication and placebo	HB, ferritin

### 2.4 Statistical analysis

This meta-analysis includes cross-sectional, cohort, and case–control studies. Eligible studies are summarized, and relevant data are presented in tables. We assessed heterogeneity using the I^2^statistic, with statistical significance of heterogeneity evaluated through a chi-square test (*p* < 0.05). The I^2^ values were interpreted as follows: 0–25% indicated no significant heterogeneity, 26–50% indicated low heterogeneity, 51–75% indicated moderate heterogeneity, and values greater than 75% suggested high heterogeneity. A fixed-effects model was used when I^2^ was less than 50%, and a random-effects model was applied when I^2^ exceeded 50%. To further explore potential sources of heterogeneity, we conducted meta-regression analyses. This allowed us to examine how study-level characteristics (e.g., study design, sample size, geographical location, or other relevant covariates) might explain variation in effect sizes across studies. Publication bias was assessed using a funnel plot, and subgroup analyses were conducted by study design (i.e., cross-sectional, cohort, and case–control studies). Sensitivity analyses were performed by excluding individual studies to assess the stability of the results and the impact on heterogeneity. In the meta-analysis of RCTs, we compared the effect sizes of changes in ferritin and hemoglobin levels. We assumed a correlation of 0.5 between baseline and follow-up ferritin and hemoglobin levels. Participants were grouped according to their treatment regimen: those who received *H. pylori* eradication therapy combined with iron supplementation were compared with those who received *H. pylori* eradication therapy alone. Additionally, participants who received both anti-*H. pylori* therapy and iron supplementation were compared with those who received iron supplementation alone. The standardized mean difference (SMD) was calculated using a random-effects model. All statistical analyses were performed using RevMan 5 (version 5.1, Cochrane Collaboration) and STATA (version 17.0, United States).

### 2.5 Risk of bias

Three independent researchers (WZT, TWT, and HJL) assessed the quality of the included studies using the Newcastle-Ottawa Quality Assessment Scale (NOS). Different criteria were applied depending on the type of study. For cohort studies, the NOS evaluation focused on several aspects, including: (1) the representativeness of the exposed cohort, (2) the appropriate selection of the non-exposed cohort, (3) the suitability of the exposure factor, (4) the clarity of outcome measures (which did not need to be observed at the study’s onset), (5) the comparability of the exposed and non-exposed groups in terms of design and statistical analysis, (6) the adequacy of outcome evaluation, (7) the duration of follow-up after the outcome, and (8) the adequacy of follow-up for both exposed and non-exposed groups. For case–control studies, the NOS evaluation included: (1) the appropriateness and representativeness of the cases, (2) the selection and appropriateness of the control group, (3) the comparability of cases and controls in terms of design and statistical analysis, (4) the accuracy of exposure factor determination, (5) the consistency of methods used to assess exposure in both cases and controls, and (6) ensuring that the non-response rate was similar between the case and control groups. The maximum score on the NOS scale was 9, with scores of 6–8 indicating good quality. For RCTs, we applied the quality assessment framework proposed by the Cochrane Collaboration. All relevant data from the included studies were systematically compiled into Tables 1, 2. Potential biases identified during the quality assessment process are also highlighted in these tables.

## 3 Results

**Figure 1:**
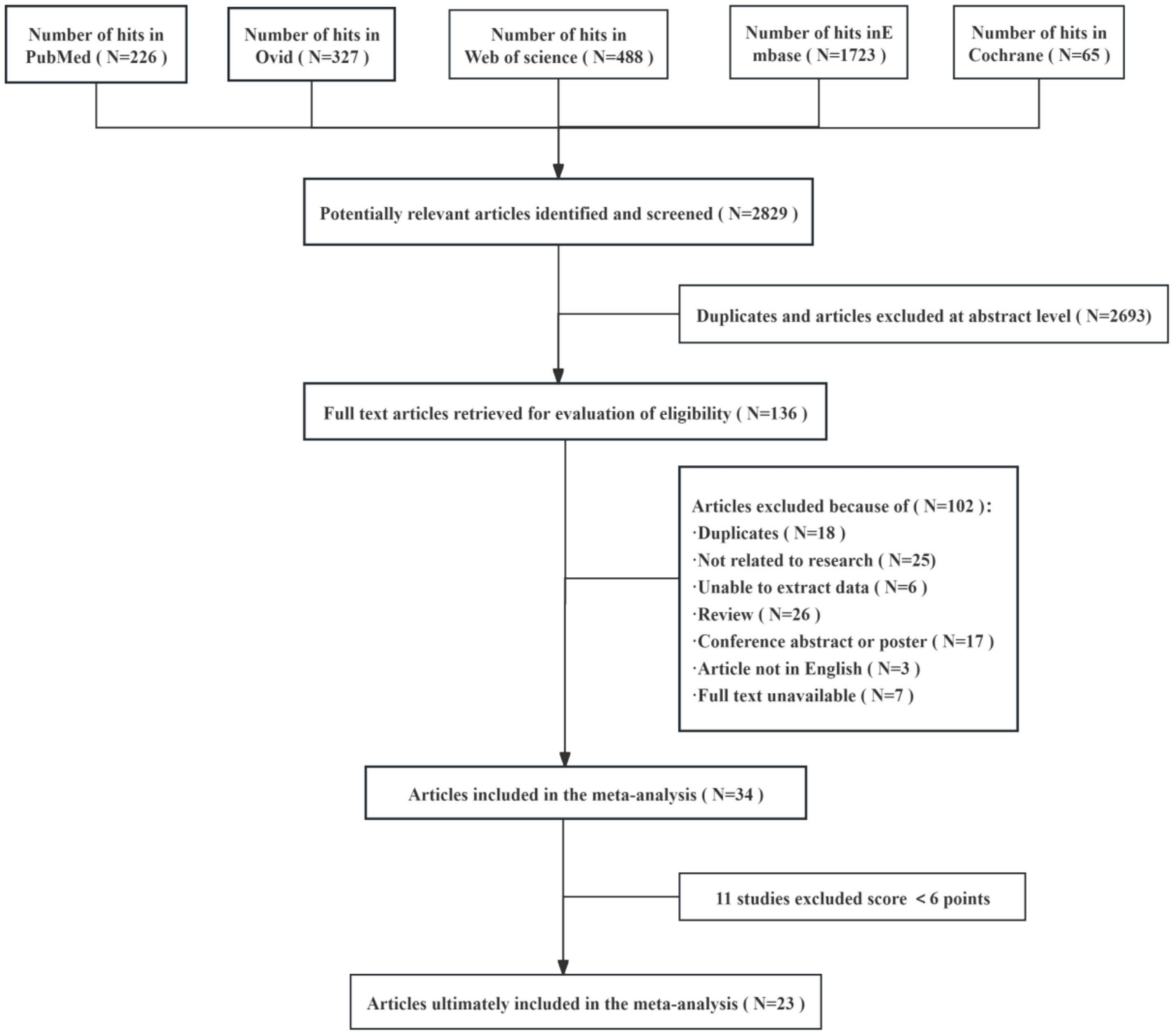
Flow diagram of the number of studies screened and included in the meta-analysis.

In this study, a total of 2,829 relevant articles were retrieved from several databases, including PubMed, Ovid, Web of Science, Embase, and Cochrane. After automatic de-duplication and screening of titles and abstracts, 2,693 duplicate or irrelevant papers were excluded, leaving 136 articles for full-text review and further screening. During the full-text screening phase, 102 articles were excluded for the following reasons: duplicates, unrelated to the research topic, unextractable data, review articles, conference abstracts or posters, non-English language, and unavailable full text. Ultimately, 34 articles were included in the meta-analysis. Following a quality assessment, 11 studies were excluded for scoring below 6 points, resulting in 23 studies being included in the final meta-analysis. This rigorous screening process ensured the scientific validity of the included studies and the reliability of the data (Figure 1).

### 3.1 *Helicobacter pylori* infection and ID

**Figure 2:**
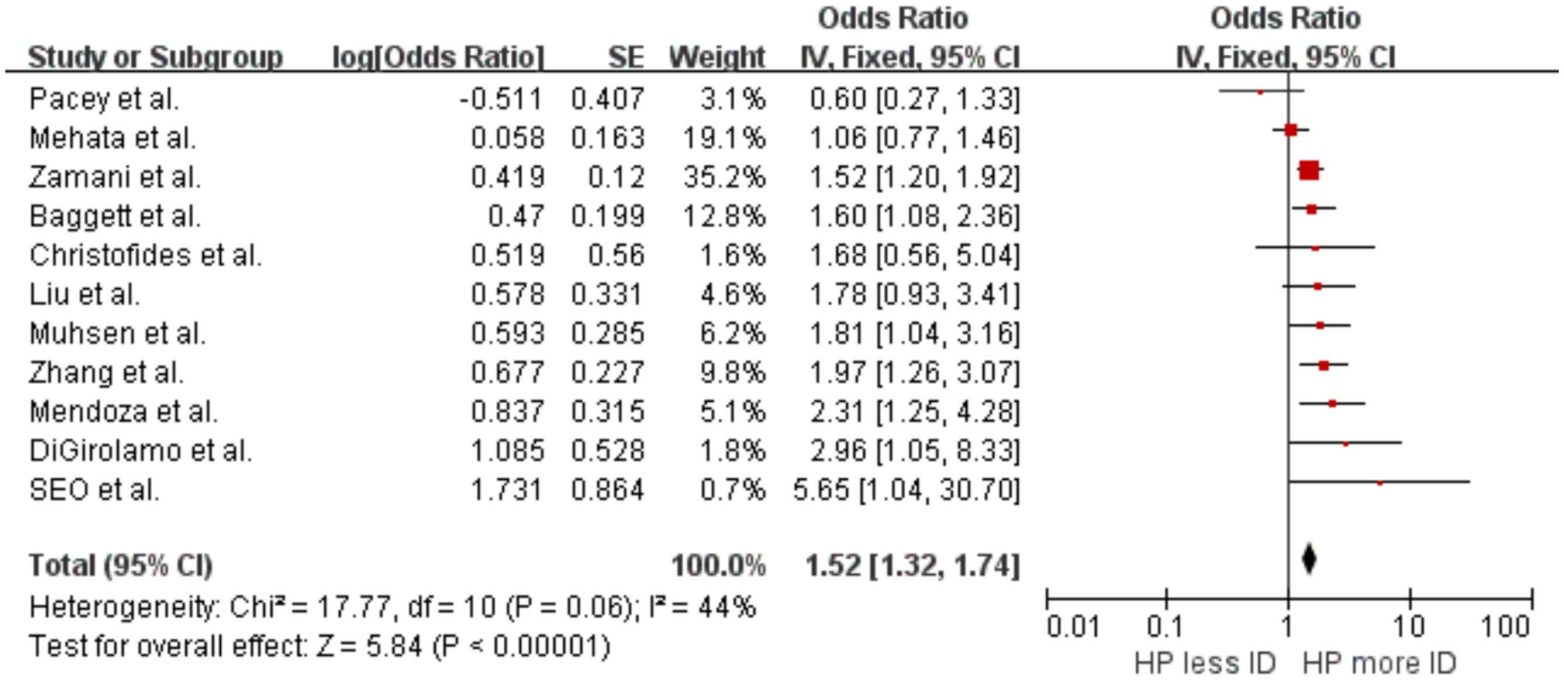
Forest plot of the meta-analysis on *H. pylori* infection and iron deficiency.

Our meta-analysis included eight cross-sectional studies (Mendoza et al., 2019; Zhang et al., 2022; Muhsen et al., 2009; Christofides et al., 2005; Pacey et al., 2011; Mehata et al., 2021; Zamani et al., 2011; Seo et al., 2002) and three clinical and interventional trial studies (Baggett et al., 2006; DiGirolamo et al., 2007; Liu et al., 2024). The results showed an increased likelihood of developing ID in individuals with evidence of *H. pylori* infection compared with uninfected individuals, with OR = 1.52 (*p* < 0.00001, 95% CI 1.32–1.74, I^2^ = 44%) (Figure 2). There was significant heterogeneity between studies, with moderate heterogeneity between studies. Sensitivity analyses suggest that the study by Mehata et al. (2021) led to this heterogeneity. Analysis excluding these studies showed no significant heterogeneity, with OR = 1.65 (*p* < 0.00001, 95% CI 1.41–1.93, I^2^ = 24%).

### 3.2 *Helicobacter pylori* infection and IDA

**Figure 3:**
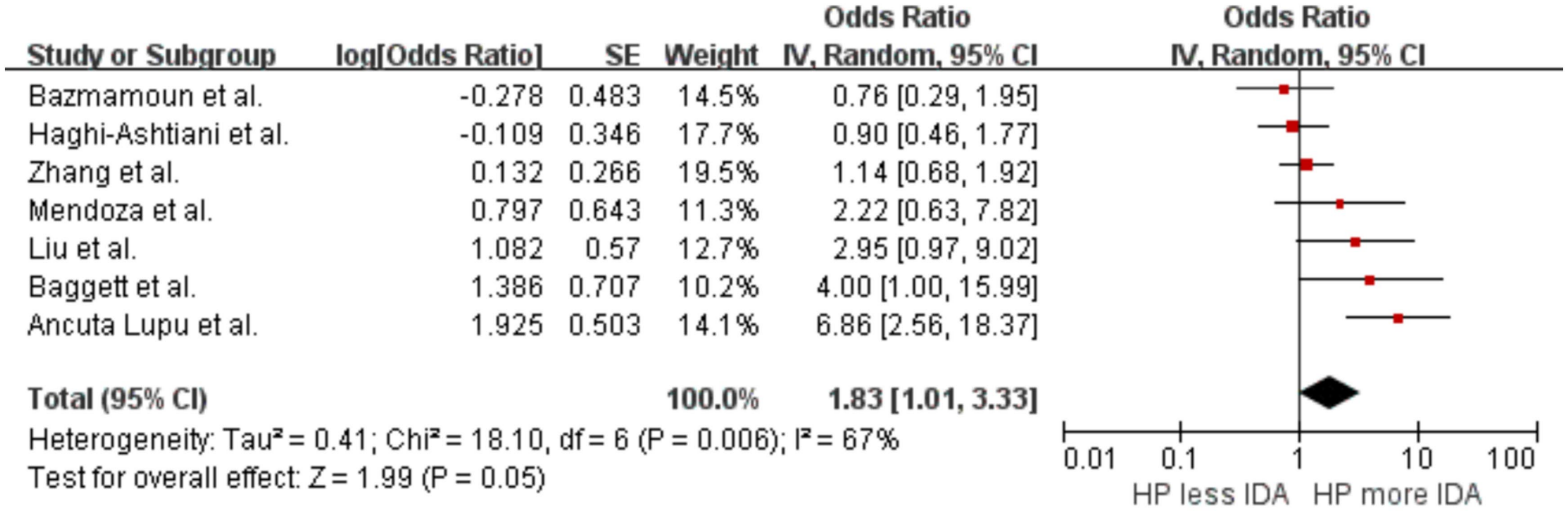
Forest plot of the meta-analysis on *H. pylori* infection and iron deficiency anemia.

A summary analysis was conducted on seven studies (Mendoza et al., 2019; Zhang et al., 2022; Baggett et al., 2006; Liu et al., 2024; Haghi-Ashtiani et al., 2008; Bazmamoun et al., 2014; Lupu et al., 2022) to explore the correlation between *H. pylori* infection and IDA. The results indicated that individuals infected with *H. pylori* had a higher likelihood of developing IDA compared to those not infected, with an OR of 1.83 (*p* = 0.05, 95% CI 1.01–3.33, I^2^ = 67%) (Figure 3). The initial analysis showed significant heterogeneity between studies. After excluding the study by Lupu et al. (2022), heterogeneity decreased, yielding an I^2^of 37%.

### 3.3 *Helicobacter pylori* infection and anemia

**Figure 4:**
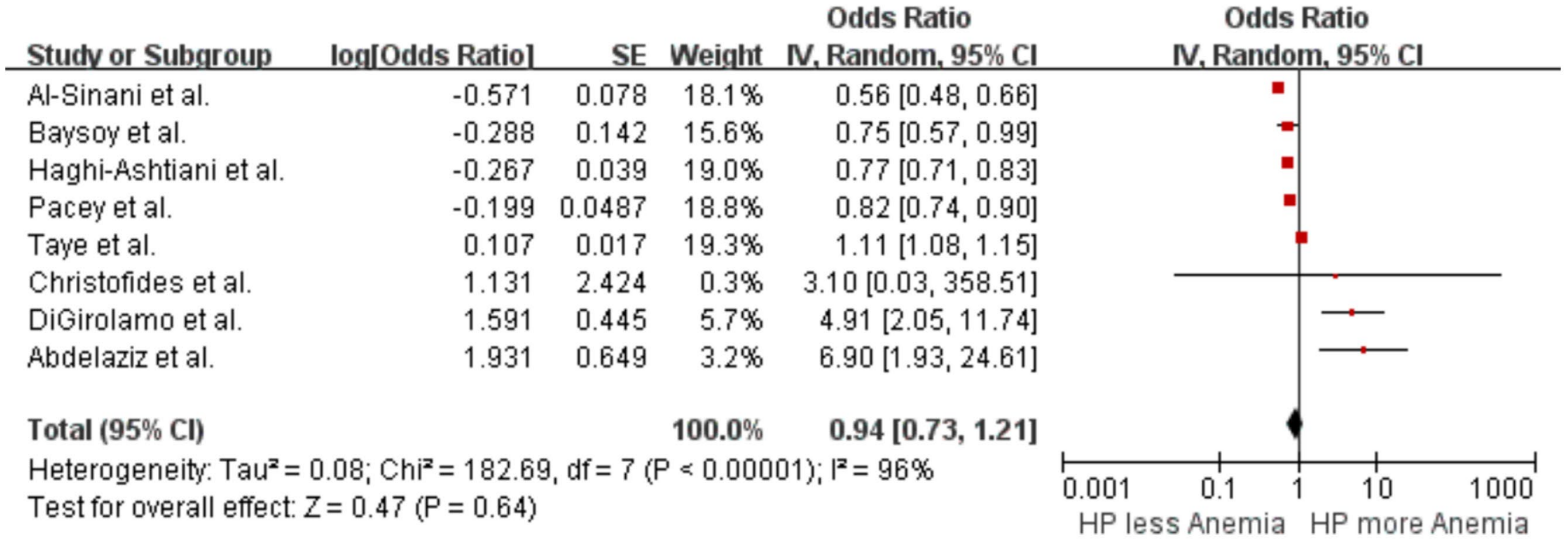
Forest plot of the meta-analysis on *H. pylori* infection and anemia.

A pooled analysis of eight studies (Christofides et al., 2005; Pacey et al., 2011; DiGirolamo et al., 2007; Haghi-Ashtiani et al., 2008; Baysoy et al., 2004; Al-Sinani et al., 2014; Abdelaziz et al., 2021; Taye et al., 2015) examined the association between *H. pylori* infection and the occurrence of anemia. The results showed no significant difference in the likelihood of anemia between patients with *H. pylori* infection and those without, with an OR of 0.94 (*p* = 0.64, 95% CI 0.73–1.21, I^2^ = 96%) (Figure 4). There was significant heterogeneity among the studies.

### 3.4 Heterogeneity analysis

To identify the sources of heterogeneity in the association between *H. pylori* infection and IDA, we conducted a meta-regression analysis considering factors such as age, gender, geographical region (developed vs. developing), and ethnicity. The results of the meta-regression indicated that age was the primary source of heterogeneity (*p* = 0.044) (Table 3). Specifically, younger individuals infected with *H. pylori* were more likely to develop IDA. This finding aligns with the objectives of our study and further supports the conclusions drawn from our analysis.

**Table 3:** Multivariable meta-regression model for moderators of *H. pylori* and iron deficient anemia.

Moderator	Coefficient	S. E.	95% C. I. low	95% C. I. high	*p*-value
Sex	3.233	6.283	−9.083	15.549	0.607
Age	0.658	0.327	0.017	1.300	**0.044**
Whether developed country	0.587	1.518	−2.389	3.562	0.699
Race	−0.243	0.809	−1.828	1.342	0.764

C. I., confidence interval; S. E., standard error; Bold indicates statistically significant.

### 3.5 Clinical trials

Currently, the commonly used methods for eradicating *H. pylori* include quadruple therapy, which consists of a PPI, two antibiotics, and a gastric mucosal protective agent. In some regions, simpler triple therapy is also employed, along with the latest medications such as Potassium-Competitive Acid Blocker. The success rate of *H. pylori* eradication is influenced by various factors, including patient resistance, medication compliance, and regional differences. Typically, the eradication rate falls within the range of 80 to 90%. Our meta-analysis additionally evaluates the impact of *H. pylori* eradication therapy on iron reserves in children. Children constitute the primary focus of our research, and the included patients are those diagnosed with ID, IDA or anemia. Our study ultimately included five RCTs (Fagan et al., 2009; Duque et al., 2010; Cardenas et al., 2011; Xia et al., 2012; Choe et al., 1999). We compare the changes in hemoglobin concentration and ferritin concentration to reflect whether receiving treatment to eradicate *H. pylori* affects the patient’s iron reserves. Participants were classified based on their treatment regimen: those who received both *H. pylori* eradication therapy and iron supplementation were compared with those who received only *H. pylori* eradication therapy. Furthermore, participants who received both anti-*H. pylori* therapy and iron supplementation were compared with those who received iron supplementation alone.

**Figure 5:**
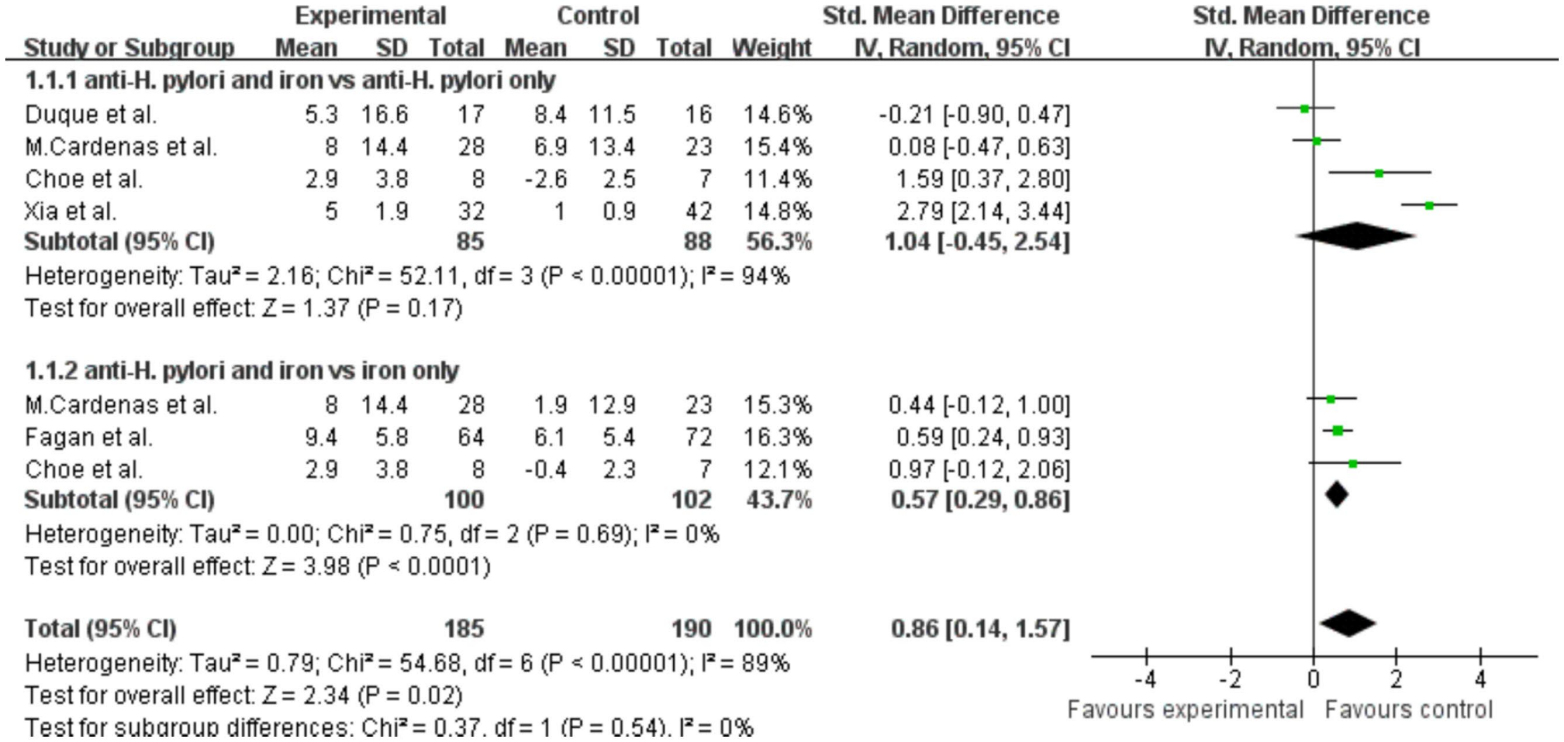
The effect of anti-*H. pylori* therapy plus iron supplementation compared to iron supplementation alone on serum ferritin levels, meta-analysis of fixed controlled trials.

Summary analysis indicated that, compared with the *H. pylori* eradication group alone, the ferritin level in the combined *H. pylori* eradication and iron supplementation group was slightly increased, SMD = 1.04 (*p* = 0.17, 95% CI −0.45–2.54, I^2^ = 94%). Significant heterogeneity was observed between the groups (Figure 5). However, when comparing the group receiving both iron therapy and *H. pylori* eradication to the subsidized treatment group alone, significant increase in ferritin levels was found, SMD = 0.57 (*p* < 0.0001, 95% CI 0.29–0.86, I^2^ = 0%). Additionally, when the two treatments were combined, there was a statistically significant difference in ferritin levels, with SMD of 0.86 (*p* = 0.02, 95% CI 0.14–1.57, I^2^ = 89%).

**Figure 6:**
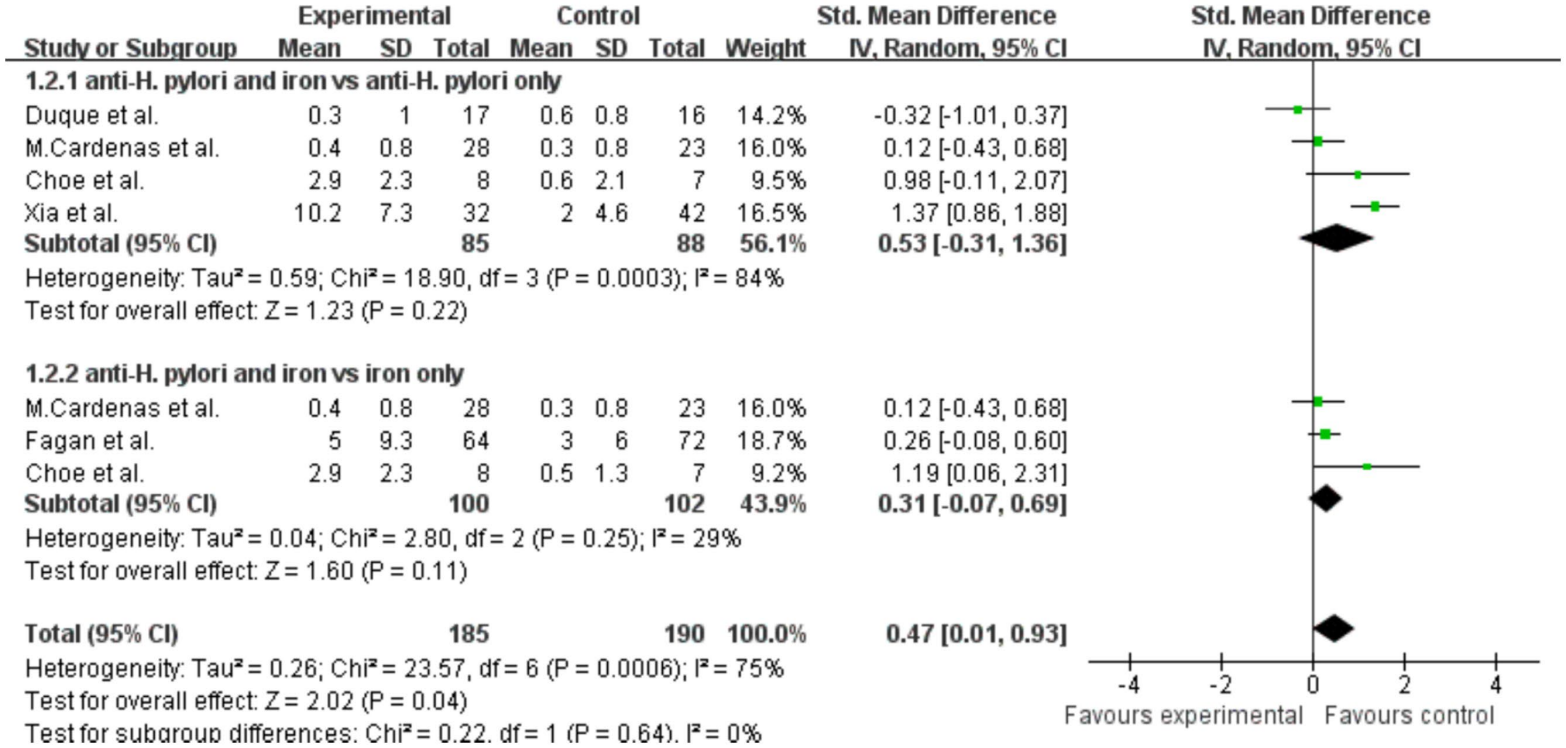
The effect of anti-*H. pylori* therapy plus iron supplementation compared to iron supplementation alone on hemoglobin levels, meta-analysis of randomized controlled trials.

Analysis of hemoglobin results showed that, compared with the *H. pylori* eradication group alone, there was no significant change in hemoglobin levels in the combined *H. pylori* eradication and iron supplementation group, SMD = 0.53 (*p* = 0.22, 95% CI −0.31–1.36, I^2^ = 84%). High heterogeneity was observed within the groups (Figure 6). Similarly, there was no significant increase in hemoglobin levels in the group receiving both iron therapy and *H. pylori* eradication compared with the group receiving iron supplementation alone, SMD = 0.31 (*p* = 0.11, 95% CI −0.07–0.69, I^2^ = 29%). Additionally, significant difference in hemoglobin levels was observed when the effects of both treatments were combined, SMD = 0.47 (*p* = 0.04, 95% CI 0.01–0.93, I^2^ = 75%).

### 3.6 Assessment of publication bias

**Figure 7:**
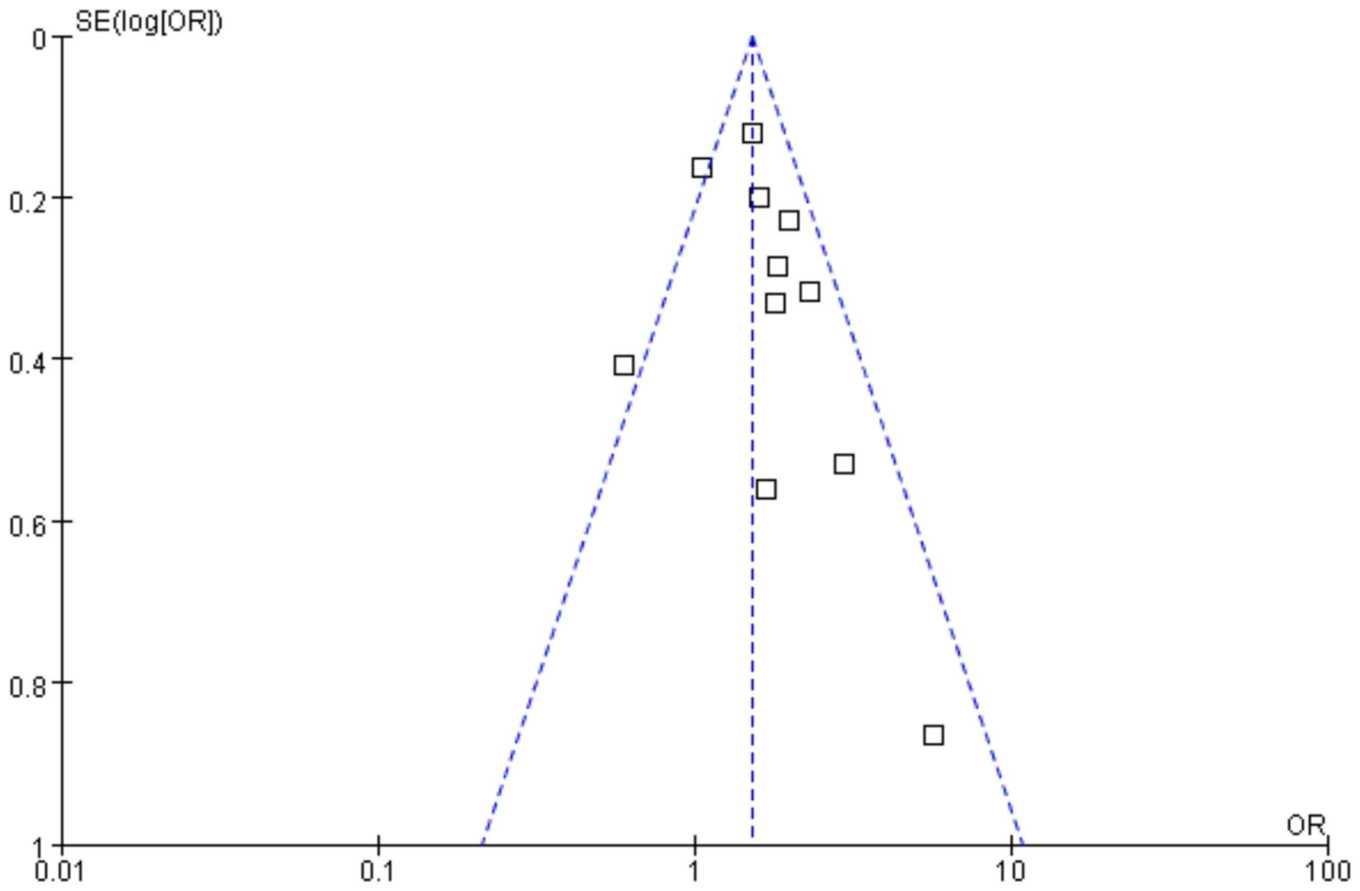
Funnel plot for event rate in the meta-analysis on *H. pylori* infection and iron deficiency.

**Figure 8:**
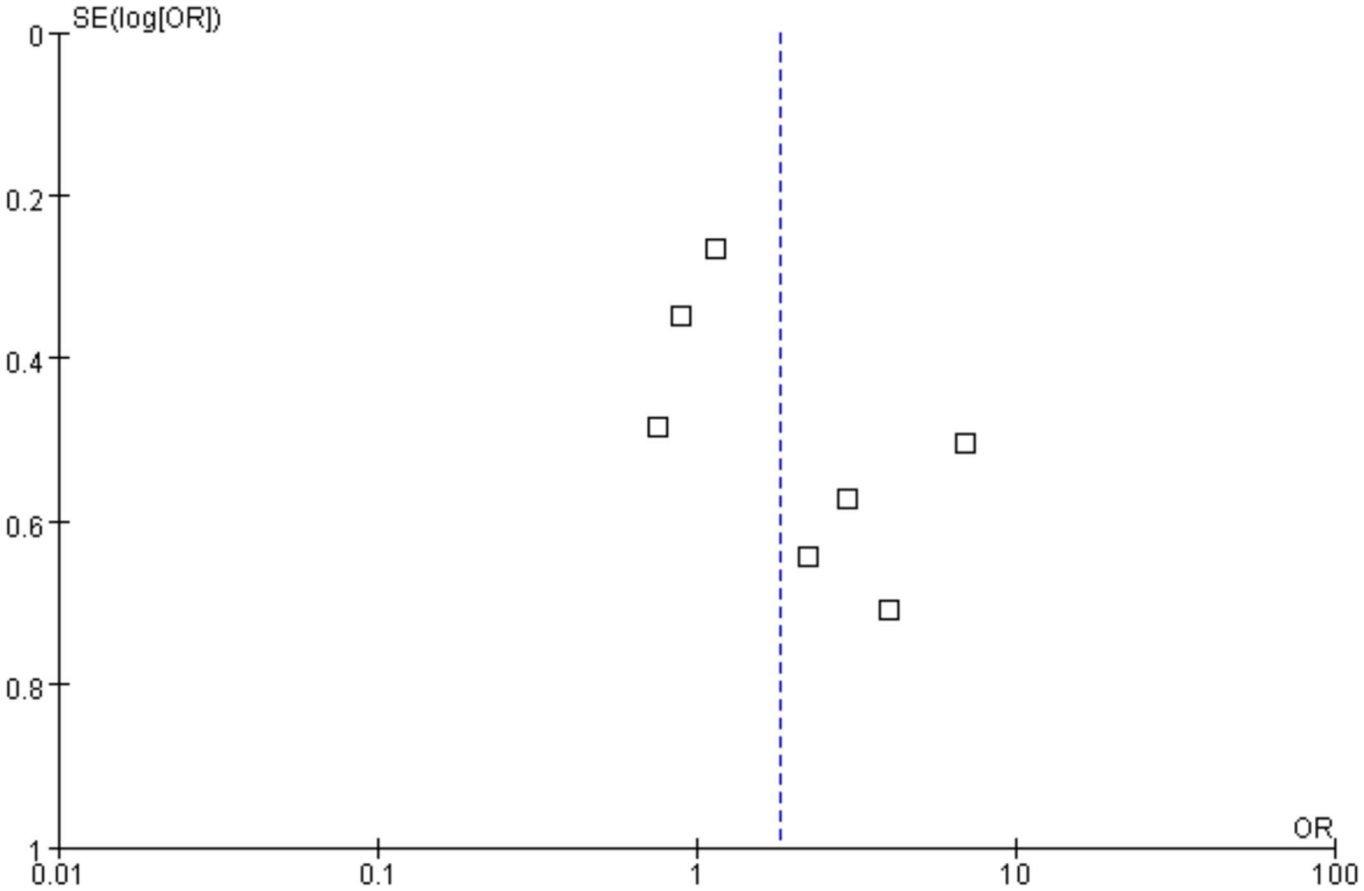
Funnel plot for event rate in the meta-analysis on *H. pylori* infection and iron deficiency anemia.

Funnels were constructed based on effect estimates and accuracy for each study to assess the presence of publication bias (Figures 7, 8). The graph is relatively symmetrical, but there are still partly favorable factors for publication.

## 4 Discussion

### 4.1 Main findings

This study systematically integrated data including a total of 13,573 pediatric patients from 16 regions, representing three ethnic groups, with ages under 20. Our core findings reveal a significant positive association between *H. pylori* infection and both ID and IDA, while no statistically significant association was found with the overall incidence of anemia. These results provide new evidence to better understand the systemic impact of *H. pylori* infection, particularly its role in iron metabolism and deficiency. In the meta-analysis of ID, the combined OR = 1.52 (95% CI 1.32–1.74), indicating a 52% increased risk of ID in individuals infected with *H. pylori* compared to uninfected individuals. Although moderate heterogeneity (I^2^ = 44%) was observed, sensitivity analysis showed that excluding the study by Mehata et al. (2021) significantly reduced the heterogeneity to I^2^ = 24%, while strengthening the effect size to OR = 1.65, further confirming the robustness of the original findings. Regarding the association with IDA, the combined OR = 1.83 (95% CI 1.01–3.33), demonstrating a stronger effect. This finding aligns with previous mechanistic studies, which suggest that *H. pylori* may exacerbate ID through multiple pathways, including impaired iron absorption due to decreased gastric acid secretion, elevated hepcidin levels induced by chronic inflammation, and bacterial iron acquisition mechanisms that deplete host iron stores. Importantly, our study found no significant association between *H. pylori* infection and overall anemia (OR = 0.94, *p* = 0.64), despite high heterogeneity (I^2^ = 96%). This apparent contradiction may stem from differences in the types of anemia included in the studies. Specifically, in non-IDA, the anemia-inducing effect of *H. pylori* may be overshadowed by other underlying causes. This highlights the importance of precise classification in anemia etiology research and suggests that the anemia-inducing mechanisms of *H. pylori* are specific to iron metabolism. In conclusion, *H. pylori* infection is significantly positively associated with IDA in children, with the effect mediated through multiple mechanisms impacting iron metabolism.

In addition, our study observed that *H. pylori* infection exerts a more indirect or less significant influence on anemia. This result is consistent with our initial expectations. We believe this is due to the varying underlying causes of anemia. Chronic anemia, for instance, is frequently associated with systemic chronic inflammation and immune responses rather than solely with disorders of iron absorption and utilization (Gallagher, 2022). In such cases, inflammatory cytokines, including tumor necrosis factor-alpha and interleukin-6, stimulate hepatic iron regulation, thereby reducing iron release and utilization (Raj, 2009). This leads to iron “sequestration” within the body, where iron reserves remain adequate, yet the iron cannot be efficiently transported to the bone marrow, ultimately resulting in anemia. Aplastic anemia (Young, 2018), on the other hand, typically arises from insufficient bone marrow hematopoietic function or abnormalities in hematopoietic stem cells. The direct relationship between local gastric inflammation caused by *H. pylori* infection and bone marrow hematopoietic function appears to be minimal, rendering *H. pylori* infection’s impact on this type of anemia relatively insignificant. Additionally, *H. pylori* infection may indirectly impair iron metabolism by altering the gut microbiota composition (Chen et al., 2021). Disruptions to intestinal health, particularly to the integrity of the intestinal barrier, could further hinder iron absorption. However, the extent of this effect on non-IDA, such as chronic anemia, remains uncertain and warrants further investigation. In summary, while *H. pylori* infection plays a critical role in the pathogenesis of IDA, its effects on other forms of anemia are more complex and often indirect. Non-iron-deficiency anemias, such as chronic anemia or aplastic anemia, frequently involve broader systemic immune responses or bone marrow dysfunction, processes that *H. pylori* infection influences only under specific conditions. Further studies are required to elucidate these nuanced interactions and better understand the broader implications of *H. pylori* infection in anemia subtypes.

To further investigate the sources of heterogeneity in the association between *H. pylori* infection and IDA, we conducted a meta-regression analysis that included potential confounders such as age, gender, region (developed vs. developing), and ethnicity. Our regression analysis revealed that age is a significant factor contributing to the observed heterogeneity. This finding aligns with our research objectives and provides further support for the strong association between *H. pylori* infection and IDA, particularly in children (Milman et al., 1998). Furthermore, our study suggests that younger patients may be more prone to developing IDA following *H. pylori* infection. There are several potential reasons for this observed relationship. First, the gastrointestinal system of young children is still developing and not yet fully matured. As such, it is more susceptible to gastric mucosal inflammation and reduced gastric acid secretion caused by *H. pylori* infection (Kostaki et al., 2003). Since iron absorption predominantly occurs in the small intestine and gastric acid plays a crucial role in the dissolution and absorption of iron, young children are at a higher risk of developing IDA due to the impaired absorption of iron (Chen et al., 2021). Second, compared to adults, children—especially infants and toddlers—have lower iron reserves. Consequently, any factors that impair iron absorption or increase iron loss will have a more pronounced effect on their iron status, making them more vulnerable to IDA (Queiroz et al., 2024). Third, the typical diet of young children, which is often primarily based on breast milk or formula, may not provide sufficient iron to meet their needs. This can further exacerbate ID (Goddard et al., 2011). Taken together, these factors suggest that young children are particularly vulnerable to the negative effects of *H. pylori* infection on iron absorption. Their developing immune and gastrointestinal systems, coupled with lower iron stores, increase their susceptibility to IDA.

Notably, our meta-analysis revealed no significant associations between *H. pylori*-IDA interactions and gender, geographic region, or ethnicity. The relationship between *H. pylori* infection and IDA exhibits regional variations influenced by socioeconomic and environmental factors. In developing countries, higher *H. pylori* prevalence (attributed to inadequate sanitation, contaminated water sources, and overcrowded living conditions) interacts synergistically with malnutrition and low dietary iron intake, exacerbating iron absorption impairments and IDA risk (Li et al., 2023). Conversely, developed nations demonstrate lower infection rates and better nutritional status, potentially mitigating *H. pylori*’s hematological consequences (Sitas et al., 1992). However, our findings do not align with the aforementioned conclusion. This apparent homogeneity may stem from three methodological considerations: First, the predominance of studies from developed regions created sampling bias, potentially underrepresenting socioeconomic heterogeneity within developing nations. Second, while infection rates remain elevated in low-income populations across all regions, this persistent exposure may diminish observable geographic variation. Third, confounding variables such as age-related physiological changes and comorbidities might overshadow regional effects. These findings underscore the necessity for population-specific interventions. Further research employing stratified sampling across socioeconomic subgroups is required to elucidate the complex interplay between environmental determinants and host factors in *H. pylori*-associated iron metabolism disorders.

Finally, we conducted a summary analysis of changes in ferritin and hemoglobin concentrations following the eradication of *H. pylori*. The results demonstrated a significant increase in ferritin levels after eradication, indicating an improvement in iron stores. However, the change in hemoglobin concentrations did not reach statistical significance, suggesting that while *H. pylori* eradication improves iron reserves, its direct impact on hemoglobin levels may be more limited. Several factors may explain this discrepancy. First, ferritin serves as the primary storage form of iron in the body and reflects the level of available iron reserves. After *H. pylori* eradication, improved iron absorption leads to a rapid restoration of iron stores, as evidenced by the increase in ferritin levels (Mahroum et al., 2022). However, the restoration of hemoglobin concentrations likely requires a longer period of iron supplementation or replenishment, especially in individuals with a history of long-term IDA (Shesh and Connor, 2023). Second, chronic gastritis resulting from *H. pylori* infection can have lasting effects on iron absorption and utilization. Even after the bacterium is eradicated, the inflammatory state of the gastric mucosa may persist for some time (Homan et al., 2024), potentially accompanied by other gastrointestinal dysfunctions. This ongoing inflammation can continue to impair the effective absorption of iron, thereby limiting hemoglobin synthesis. Finally, while ferritin levels reflect iron storage, the distribution and utilization of iron in the body are regulated by complex mechanisms, including hepcidin, a key regulator of iron homeostasis (Yanatori et al., 2023). After *H. pylori* eradication, iron absorption may improve rapidly, but the redistribution and utilization of iron for processes like hemoglobin synthesis could take longer to fully reflect. This delayed redistribution may explain why hemoglobin concentrations take more time to respond to the improved iron availability (Zhang et al., 2021; Sharndama and Mba, 2022; Carabotti et al., 2021). In summary, following the eradication of *H. pylori*, ferritin levels showed a significant increase, which provides further evidence that early eradication of *H. pylori* can help improve iron stores in the body, particularly in children. This finding suggests that addressing *H. pylori* infection early may play a crucial role in restoring iron reserve levels and mitigating the risk of IDA in affected populations.

### 4.2 Advantages of the meta-analysis

This paper has several notable strengths. It provides a comprehensive review of the relationship between *H. pylori* infection and ID, IDA, and other types of anemia, systematically analyzing potential mechanisms and impacts. By focusing on the pediatric population, it offers valuable insights into an important yet underexplored group. Additionally, the inclusion of studies from diverse regions enhances the generalizability of the findings and highlights differences in healthcare systems and nutritional statuses across populations. From a mechanistic perspective, the paper explores how *H. pylori* infection contributes to ID and IDA through its effects on gastric acid secretion, iron absorption, iron metabolism, and gastric mucosal damage. This in-depth analysis advances the understanding of its pathogenic mechanisms. Moreover, the study underscores the importance of early diagnosis and intervention, providing practical guidance for clinical practice and a foundation for future prevention and treatment strategies.

### 4.3 Limitations of the meta-analysis

This study highlights a link between *H. pylori* infection and IDA but has several limitations that warrant further investigation. First, it primarily focuses on the pediatric population, leaving the effects on adults, the elderly, and individuals with chronic conditions underexplored. Second, the absence of bone marrow puncture biopsy, the gold standard for diagnosing ID, may have affected diagnostic accuracy due to its invasive nature. Future studies should address these gaps by increasing sample diversity, utilizing more sensitive and non-invasive diagnostic methods, and considering multi-dimensional influencing factors. Additionally, the moderate quality of included studies, as indicated by NOS scores, underscores the need for well-designed RCTs. These should assess the long-term efficacy and potential risks of *H. pylori* eradication therapy for IDA. Placebo-controlled trials could clarify the direct impact of eradication on IDA recovery, but ethical concerns must be considered, as *H. pylori* infection is strongly linked to severe gastrointestinal diseases. Balancing ethical considerations with the need for robust evidence is crucial for advancing our understanding of *H. pylori* and its role in IDA and related conditions.

## 5 Conclusion

We conducted a meta-analysis using data from over 10,000 children under the age of 20 from 13 countries and 3 ethnicities to assess the relationship between *H. pylori* infection and ID as well as anemia. The results show that individuals infected with *H. pylori* have a significantly higher likelihood of developing ID compared to those uninfected. Additionally, there is a significant association between *H. pylori* infection and the occurrence of IDA, with an OR of 1.83 (*p* = 0.05, 95% CI 1.01–3.33, I^2^ = 67%). However, the overall effect of *H. pylori* infection on anemia is minimal. Age is the main source of heterogeneity. In the meta-analysis of treatment-related RCTs, the combination of iron supplementation and *H. pylori* eradication therapy significantly increased ferritin levels. Although hemoglobin levels also showed an increase, the increase was not as significant as that in ferritin levels. These results indicate a significant association between *H. pylori* infection and both ID and IDA, and that *H. pylori* eradication therapy has a certain effect on improving iron stores.

## Data Availability

The datasets presented in this study can be found in online repositories. The names of the repository/repositories and accession number(s) can be found in the article/Supplementary material.
