# Supplementary material for: Impact of Helicobacter pylori infection on iron deficiency anemia in children: a systematic review and meta-analysis with early intervention implications
Source: Front Microbiol. 2025 Jun 19;16:1541011. doi: 10.3389/fmicb.2025.1541011 (PMC12222266; doi:10.3389/fmicb.2025.1541011)
Supplement: Supplementary file 1 [file Table_1.DOCX]

**Appendix 1. Sample of search strategies (Pubmed results 09 August 2024).**

| Search number | Query | Sort  By | Filters | Search Details | Results | Time |
| --- | --- | --- | --- | --- | --- | --- |
| 1 | ("ferritin"[Title/Abstract] OR "hemoglobin"[Title/Abstract] OR "iron stores"[Title/Abstract] OR ("iron deficiency anemia"[Title/Abstract] OR "Anemia"[Title/Abstract] OR "Iron-Deficiency"[Title/Abstract] OR "iron deficiency anemia"[Title/Abstract] OR "Anemias"[Title/Abstract] OR "Iron-Deficiency"[Title/Abstract] OR "anemias iron deficiency"[Title/Abstract] OR "anemias iron deficiency"[Title/Abstract] OR "iron deficiency anemias"[Title/Abstract] OR "iron deficiency anemias"[Title/Abstract])) AND ("Child"[Title/Abstract] OR "Children"[Title/Abstract]) AND ("helicobacter pylori"[Title/Abstract] OR "helicobacter nemestrinae"[Title/Abstract] OR "campylobacter pylori"[Title/Abstract] OR ((("helicobacter pylori"[MeSH Terms] OR ("Helicobacter"[All Fields] AND "pylori"[All Fields]) OR "helicobacter pylori"[All Fields] OR ("Campylobacter"[All Fields] AND "pylori"[All Fields]) OR "campylobacter pylori"[All Fields]) AND ("subsp"[All Fields] OR "subsps"[All Fields])) AND "pylori"[Title/Abstract]) OR "campylobacter pyloridis"[Title/Abstract]) |  |  | ("ferritin"[Title/Abstract] OR "hemoglobin"[Title/Abstract] OR "iron stores"[Title/Abstract] OR ("iron deficiency anemia"[Title/Abstract] OR "Anemia"[Title/Abstract] OR "Iron-Deficiency"[Title/Abstract] OR "iron deficiency anemia"[Title/Abstract] OR "Anemias"[Title/Abstract] OR "Iron-Deficiency"[Title/Abstract] OR "anemias iron deficiency"[Title/Abstract] OR "anemias iron deficiency"[Title/Abstract] OR "iron deficiency anemias"[Title/Abstract] OR "iron deficiency anemias"[Title/Abstract])) AND ("Child"[Title/Abstract] OR "Children"[Title/Abstract]) AND ("helicobacter pylori"[Title/Abstract] OR "helicobacter nemestrinae"[Title/Abstract] OR "campylobacter pylori"[Title/Abstract] OR (("helicobacter pylori"[MeSH Terms] OR ("Helicobacter"[All Fields] AND "pylori"[All Fields]) OR "helicobacter pylori"[All Fields] OR ("Campylobacter"[All Fields] AND "pylori"[All Fields]) OR "campylobacter pylori"[All Fields]) AND ("subsp"[All Fields] OR "subsps"[All Fields]) AND "pylori"[Title/Abstract]) OR "campylobacter pyloridis"[Title/Abstract]) | 226 | 9:14:29 |

**Appendix 2. Sample of search strategies (Ovid results August 08 2024).**

Ovid MEDLINE(R) and Epub Ahead of Print, In-Process, In-Data-Review & Other Non-Indexed Citations, Daily and Versions <1946 to August 08, 2024>

1 Helicobacter pylori.af. 51837

2 Helicobacter nemestrinae.af. 7

3 Campylobacter pylori.af. 920

4 "Campylobacter pylori subsp. pylori".af. 1

5 Campylobacter pyloridis.af. 176

6 1 or 2 or 3 or 4 or 5 52759

7 Iron Deficiency Anemias.af. 121

8 Anemias.af. 4598

9 Iron Deficiency.af. 28741

10 Anemia.af. 211594

11 ferritin.af. 36866

12 Iron.af. 280354

13 hemoglobin.af. 194879

14 iron stores.af. 4235

15 7 or 8 or 9 or 10 or 11 or 12 or 13 or 14 625149

16 Child.af. 2635261

17 children.af. 1790582

18 16 or 17 3258637

19 6 and 15 and 18 327

**Appendix 3. Sample of search strategies (WOS results August 09, 2024).**

| Right | # | Serch | Data | results | time |
| --- | --- | --- | --- | --- | --- |
| - WOS.IC: 1993 to 2024  - WOS.CCR: 1985 to 2024  - WOS.SCI: 1996 to 2024  - WOS.AHCI: 1996 to 2024  - WOS.ESCI: 2019 to 2024  - WOS.ISTP: 2002 to 2024  - WOS.SSCI: 1996 to 2024  - WOS.ISSHP: 2002 to 2024 | 1 | (((((((((((ALL=(ferritin)) OR ALL=(hemoglobin)) OR ALL=(iron stores)) OR ALL=(iron deficiency anemia)) OR ALL=(Anemia)) OR ALL=(Iron-Deficiency)) OR ALL=(iron deficiency anemia)) OR ALL=(Anemias)) OR ALL=(Iron-Deficiency)) OR ALL=(anemias iron deficiency)) OR ALL=(iron deficiency anemias)) OR ALL=(iron deficiency anemias) | Web of Science Core Collection | 334928 | Fri Aug 09 2024 21:27:09 GMT+0800 (China Standard Time) |
| - WOS.IC: 1993 to 2024  - WOS.CCR: 1985 to 2024  - WOS.SCI: 1996 to 2024  - WOS.AHCI: 1996 to 2024  - WOS.ESCI: 2019 to 2024  - WOS.ISTP: 2002 to 2024  - WOS.SSCI: 1996 to 2024  - WOS.ISSHP: 2002 to 2024 | 2 | (ALL=(Children)) OR ALL=(child) | Web of Science Core Collection | 2310700 | Fri Aug 09 2024 21:27:38 GMT+0800 (China Standard Time) |
| - WOS.IC: 1993 to 2024  - WOS.CCR: 1985 to 2024  - WOS.SCI: 1996 to 2024  - WOS.AHCI: 1996 to 2024  - WOS.ESCI: 2019 to 2024  - WOS.ISTP: 2002 to 2024  - WOS.SSCI: 1996 to 2024  - WOS.ISSHP: 2002 to 2024 | 3 | (((((((((ALL=(helicobacter pylori)) OR ALL=(helicobacter nemestrinae)) OR ALL=(campylobacter pylori)) OR ALL=(helicobacter pylori)) OR ALL=(Helicobacter)) OR ALL=(helicobacter pylori)) OR ALL=(Campylobacter)) OR ALL=(campylobacter pylori)) OR ALL=(pylori)) OR ALL=(campylobacter pyloridis) | Web of Science Core Collection | 102515 | Fri Aug 09 2024 21:28:47 GMT+0800 (China Standard Time) |
| - WOS.IC: 1993 to 2024  - WOS.CCR: 1985 to 2024  - WOS.SCI: 1996 to 2024  - WOS.AHCI: 1996 to 2024  - WOS.ESCI: 2019 to 2024  - WOS.ISTP: 2002 to 2024  - WOS.SSCI: 1996 to 2024  - WOS.ISSHP: 2002 to 2024 | 4 | #1 AND #2 AND #3 | Web of Science Core Collection | 488 | Fri Aug 09 2024 21:29:03 GMT+0800 (China Standard Time) |

**Appendix 4. Sample of search strategies (cochrane results August 09, 2024).**

Search Name:

Date Run: 09/08/2024 15:46:10

Comment:

ID Search Hits

#1 ferritin 5886

#2 hemoglobin 47339

#3 iron stores 946

#4 iron deficiency anemia 4397

#5 Anemia 26692

#6 Iron-Deficiency 4846

#7 iron deficiency anemia 4397

#8 Anemias 230

#9 Iron-Deficiency 4846

#10 anemias iron deficiency 74

#11 anemias iron deficiency 74

#12 iron deficiency anemias 74

#13 iron deficiency anemias 74

#14 #1 or #2 or #3 or #4 or #5 or #6 or #7 or #8 or #9 or #10 or #11 or #12 or #13 68244

#15 Child 214019

#16 children 214019

#17 teenager 181

#18 #15 or #16 or #17 214082

#19 helicobacter pylori 6317

#20 helicobacter nemestrinae 0

#21 campylobacter pylori 113

#22 helicobacter pylori 6317

#23 Helicobacter 6442

#24 pylori 6963

#25 helicobacter pylori 6317

#26 Campylobacter 407

#27 campylobacter pylori 113

#28 campylobacter pyloridis 28

#29 #19 or #20 or #21 or #22 or #23 or #24 or #25 or #26 or #27 or #28 7380

#30 #14 and #18 and #29 65

**Appendix 5. Sample of search strategies (Embase results August 09, 2024).**

**Embase session results (9 Aug 2024)**

| No. | Query | Results |
| --- | --- | --- |
| #31 | #13 AND #17 AND #30 | 1723 |
| #30 | #18 OR #19 OR #20 OR #21 OR #22 OR #23 OR #24 OR #25 OR #26 OR #27 OR #28 OR #29 | 239895 |
| #29 | hp | 129506 |
| #28 | h.pylori | 46526 |
| #27 | campylobacter AND pyloridis | 203 |
| #26 | campylobacter AND pylori | 2894 |
| #25 | campylobacter | 28022 |
| #24 | helicobacter AND pylori | 81456 |
| #23 | pylori | 84702 |
| #22 | helicobacter | 90064 |
| #21 | helicobacter AND pylori | 81456 |
| #20 | campylobacter AND pylori | 2894 |
| #19 | helicobacter AND nemestrinae | 15 |
| #18 | helicobacter AND pylori | 81456 |
| #17 | #14 OR #15 OR #16 | 4202827 |
| #16 | teenage | 13963 |
| #15 | children | 2526127 |
| #14 | child | 3361569 |
| #13 | #1 OR #2 OR #3 OR #4 OR #5 OR #6 OR #7 OR #8 OR #9 OR #10 OR #11 OR #12 | 1002334 |
| #12 | iron AND deficiency AND anemias | 1374 |
| #11 | anemias AND iron AND deficiency | 1374 |
| #10 | 'iron deficiency' | 62807 |
| #9 | anemias | 6486 |
| #8 | iron AND deficiency AND anemia | 52331 |
| #7 | 'iron deficiency' | 62807 |
| #6 | anemia | 473670 |
| #5 | iron AND deficiency AND anemia | 52331 |
| #4 | ferritin | 85307 |
| #3 | iron AND stores | 7472 |
| #2 | hemoglobin | 574807 |
| #1 | 'ferritin'/exp OR ferritin | 85307 |
